# “Poster girl”: The discourse constructing the image of “girls in distress” as existential epistemic injustice

**DOI:** 10.3389/fpsyt.2022.966778

**Published:** 2022-11-15

**Authors:** Lia Levin, Maya Cohen Brafman, Raghda Alnabilsy, Shira Pagorek Eshel, Haneen Karram-Elias

**Affiliations:** ^1^Bob Shapell School of Social Work, Tel Aviv University, Tel Aviv, Israel; ^2^Department of Social Work, Ruppin Academic Center, Emek Hefer, Israel

**Keywords:** girls, distress, social services, policy, epistemic justice, discourse

## Abstract

The present study is focused on understanding how the image of the girl designated “in distress” in official regulations guiding the provision of public social services to girls in Israel can be structured. The study takes a qualitative approach, and employs the critical-feminist paradigm to the analysis and interpretation of discourse, combining thematic content analysis and deductive critical discourse analysis. Its main findings disclose an organized process of establishing the normative authorities dominating the discourse on public social services for girls; classifying groups of service recipients to which a girl can belong; constructing their forms; and ultimately circumscribing the girls thereto, determining the performative acts on which receiving state assistance is conditional. Through discursive maneuvers of construction, the image of the girl is “born” as an undisputed “truth” deriving from the deviance attached to her every move. In this trajectory, basic epistemic injustices are perpetuated and solidified, and a new form of epistemic injustice—existential epistemic injustice—is revealed. This process's implications are proposed.

## Introduction

“Language” is defined as “the words, their pronunciation, and the methods of combining them used and understood by a community” (1). According to the Sapir-Whorf hypothesis, language is subconsciously secured in societies' communicative and interpretative habits (2). As such, language and its rules play an important part in establishing social and group identities. A language's construction echoes the aspiration for finding order in social structures, and is often regulated by those who possess the power to control its content (3). “Discourse”, constitutes the practice of conceptualizing and exchanging ideas using language (4).

The present study addresses the construction of the image of girls in need of assistance from Israeli public social services, as it is reflected in the language and discourse prevalent in state regulations guiding such services. These girls are referred to in Hebrew using the term *na'arót beMetzuká* [“girls in distress”]. Our research approach can be identified as critical-feminist, incorporating ideas of Michel Foucault (5–8) and Judith Butler (9–11), insofar as these address the structural role of language and discourse in the evolution of gender forms and the social tasks assigned thereto. The study rests on the supposition, suggested as shared by both Foucault and Butler, by which there is no human depiction with an “identity” or “essence” that precedes discourse, and that any implied existence thereof is in fact the result of structural mechanisms, a central component of which is language. As will be shown, these assumptions will be applied toward gaining better understandings of the epistemic justice underpinnings of discourse practices, in general and with specific relation to girls dealing with distress.

According to Foucault (5, 6), power involved in acting, creating, and perpetuating social orders is applied through interventions in the lives of individuals, in ways that (1) subject individuals to the examination and surveillance of others who control the discourse; (2) shape what is assessed as individuals' social “particularity”; and (3) forcibly tie individuals to the particularity that determines their position within the oppressive social structure. Such power simultaneously bears disciplining and manufacturing attributes, and is definitively external to the subjects at whom it is directed. A prominent mechanism executing this power is *form*, which “categorizes the individual, marks him by his own individuality, attaches him to his own identity, imposes a law of truth on him which he must recognize and which others have to recognize in him” [(7), p. 781]. So, *via* formative power, the individual takes on distinctive qualities, or definitions, that set her/him apart, that are integrated into her/his being and body, become an inseparable part of her/his existence, and are present in all of her/his actions (8).

Foucault (5) illustrates the inseparability of knowledge/power through the space of inspection. The power and control inflicted by inspection are often concealed, and are enabled through the acquisition of the status of rationality, objectivity and science. Accordingly, inspection becomes an antecedent of applying ritual and “scientific” actions to “fix” individuals based on the differences between them; and at the same time, these differences are prioritized, measured, “marked”, and categorized by whomever controls discipline, thus reducing individuals to the traits that are inspected, or to mere faceless “cases”. As with Foucault, institutions tasked with screening, classifying, and processing such “cases” (including prisons, schools, hospitals, and social services) are intended to create order among individuals by deeming them rational or irrational human material, worthy or unworthy of membership in the orderly world (12).

These processes run yet deeper within the social structure, as the predominance of the voice of what are established as scientific or professional authorities also delegitimizes interpretations of experts by lived experience, exacerbating testimonial epistemic injustices [discounting another's credibility based on bias toward the social groups to which she/he belongs, (13)]. In this sense, the distinction between rational and irrational, sane or insane, becomes grounds for discrediting the voice as well as the speaker. The particularities tied to individuals that are subjected to dominant discourse through processes of intervention, diagnosis, and inspection, obscure diverse identities (14). Individual accounts are devalued, based on predefined particularities that justify epistemic injustice as necessary for upholding the social order or even for providing effective treatment to those who'do not know what is good for them', and hence have little knowledge to offer to those tasked with assisting them (15). Regarding social structures and individual identities, Butler (16) contended that identities are neither natural nor static. They obtain social meaning only when repeatedly reenacted within the limits designated for them. For example, in *Gender Trouble*, Butler (9) claimed that gender is constituted as corporeal style, while it is in fact no more than a set of repeated performative actions, falsely creating the appearance of a suspended “natural” and “inalterable” fact. In this process, “material bodies” that matter are those sustained by specific appearances of sexuality marking bodies as socially comprehensible. In this vein, becoming understood socially entails obtaining meaning through systems of cultural signs (10). This means that there are no “material bodies” whose definition is not influenced by preceding cultural discourse (9). In terms of discourse, the “material body” is treated as a passive subject, marked by cultural forces external thereto. The forms inscribed onto the body, that are the consequence of the literal acts, sketch and delineate its acceptable boundaries. Butler argued that this does not mean that material bodies do not exist prior to inscription, but rather that materials and the social markings imposed thereupon are intertwined. The dominance and control over discourse thus becomes coupled with the privilege to create “social reality”, and attach appearances of meaning to existing structure and form (10).

This “social reality” suggestibly constitutes exceedingly fertile grounds for the development of hermeneutical epistemic injustices [the broad societal difficulty to understand social groups' experiences, due to the continued exclusion of members of such groups from mainstream meaning-making process; (13)]. Not only do performative acts perpetuate existing epistemic hierarchies, but they also limit opportunities for systems to develop mechanisms needed to support virtuous hearing (17). In the absence of such opportunities, contestation against dominant discourse is easily brushed aside, and the language prevalent in systems becomes so organic to their functioning, that the power relations and epistemic injustices that underlie it become difficult to identify, and even more so contest (18). Policy can play a vital role in maintaining, shaping, or correcting epistemic injustices. Policy documents often fulfill a dual aim in this respect—they both reflect current dominant discourse and solidify it by turning discursive norms into written rules (19).

Resting on the above described conceptual frameworks, and in line with the suggestion that policy documents are telling and influential objects of research when it comes to the analysis of discourse surrounding marginalized populations (20), our research centered around two main questions: What is the image of “the girl in distress” that is reflected in official regulations guiding public social services for girls in Israel? How do the language and discourse constructing regulations and constructed therein delimit the status, essence, and presence of “girls in distress” in the public sphere?

While the study is anchored in the Israeli policy context, social services and the Israeli welfare state share their distinctive attributes with several other welfare states around the world [e.g., the United Kingdom, the United States, countries in southern Europe and certain areas in the Middle East; (21)], and consequent similarities characterize the main responses and treatment afforded to assist girls considered at-risk by public social services in these countries. This renders the study's findings, as well as insights attached to its methodology, plausibly highly transferable, as well as useful and thought-provoking, to other contexts as well.

## Materials and methods

### Sample of regulations and procedure of collection

All 23 State Social Work Regulations pertaining to public social services provided to girls in Israel were analyzed. State Social Work Regulations are intended to explain and organize the legal aspects of providing public social services in Israel. In them, are concentrated the official policies guiding services and shaping their nature, scope, and practice principles, as well as information about the procedures needed to apply policy and other institutional requirements attached to offering social services through departments of social services. In the absence of an up-to-date welfare services law in Israel and/or a defined basket of personal social services that the state of Israel is obligated to offer Israeli citizens, the State Social Work Regulations are the most pertinent documents that guide services in all areas of public welfare. They are divided into 20 chapters, all of which were screened for relevant content. State Social Work Regulations are publicly accessible online, thus no authorizations were required to gather or analyze them. The regulations analyzed for the present study were all published between 1987 and 2017.

### Process of analysis

Two methods of data analysis were chosen as appropriate for achieving the aims of the present study. The first was Thematic Content Analysis (TCA). TCA is a qualitative method employing strategies of systematic coding and categorizations of textual data, and is aimed at uncovering patterns in the use of certain words, the frequency of their appearance, and the relations between words and the discourse construct that they represent (22). To that end, all regulations were read and reread several times, and comments and remarks were attached thereto. In the initial readings, marking and commenting were intuitive. In later readings, categories revealed themselves and the texts were divided according thereto.

Following this, in the second stage of analysis, Fairclough's (23) principles of Critical Discourse Analysis (CDA) were employed to examine the veiled roles of language and discourse in constructing power relations and establishing the social status of the image of the “girl in distress”. CDA is based on approaches to “discourse of power” and “discourse of racism” (24) that explain phenomena through the associations between discourse, power, oppression, and discrimination (25), and enable revealing possible outcomes of discourse in terms of creating or eroding solidarity (26). In the present study, CDA-associated deductive interpretation was guided by theoretical principles, described in the introduction, proposed by Foucault (5–8) and Butler (9, 10).

Namely, special attention was given to Foucault's (5, 6) three modalities of objectification, considered by many [e.g., (27, 28)] to be most closely related to the perpetuation of epistemic injustices: *subjectification*, through which dominant authorities who control discourse are established as normative, as are the roles and privileges attached thereto; *dividing practices* setting the rules of discourse that ensure the preservation of power by preventing the entry of “foreign” discourses into it, e.g., by deeming anything outside dominant “truths” as false; and *scientific classification*, by which idiosyncratic meaning is imposed on individuals, and actions are taken to sustain its uniformity.

To expose the specific qualities inscribed upon the image of the “girl in distress” in regulations and discuss the consequent implications of this inscription with regard to epistemic in/justices, a focus was also placed on Butler's idea of *discourse and performance*. In this vein, an attempt was made to track the construction of the gender image of girls within its cultural-political context and the form wherein the contours of this image are delimited; to identify processes of constructing the boundaries of sex and gender by stipulating compelled repeated performances; and to examine how repetitiveness contributes to the determination of the bounds of girls' images and social roles.

In practice, CDA was carried out by reading all of the texts once more, but this time from an interpretative-theoretical perspective. This resulted in a new set of categories, relating to roles, power relations and social positions.

## Results

**Figure 1:**
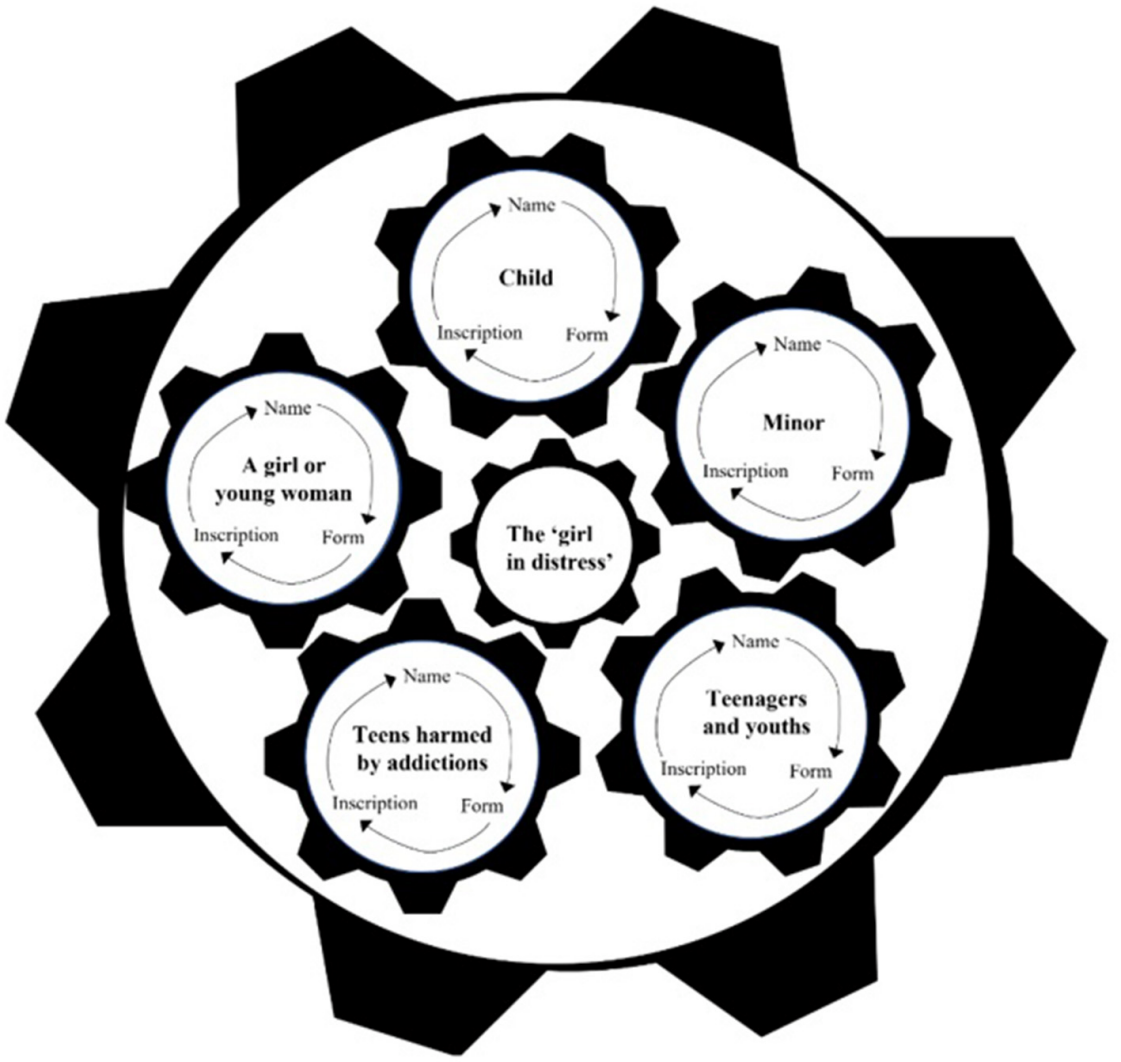
The “Machine.”

To illustrate the process of discursive construction unveiled in the present study, we chose to use an analogy of a machine (Figure 1).

The machine is comprised of seven cogwheels, rotating alongside each other, interlocked and interdependent for movement. The “machine” is bordered by an exterior wheel that symbolizes the discourse space of public social services for girls. Rotating within it are five wheels, each signifying a distinct discourse space attached to an individual type of service recipient that the “girl in distress” might be. These spaces are in turn bordered by names given to recipients of public social services, either inter alia or specifically, to girls. In each, the girl is labeled (explicitly or implicitly) as a beneficiary of assistance. Within these discourse spaces, the normative form of the image of service recipients becomes set, as does that of other figures related to it in the space of service extension.

In the center of the machine, as a byproduct of the rotating motion, lies the innermost cogwheel. While this wheel turns as a result of the movement of the wheels around it, at the same time, it is charged with keeping them turning. This wheel, that depicts the full representation of the “girl in distress”, is created from the content of the surrounding wheels, and at the same time is expressed in them. The following describes the motion of the machine vis-à-vis each of its wheels and the process of construction that they produce and sustain.

### Delineating the discourse space wherein normative authorities operate

The exterior wheel that the metaphorical “machine” borders on, is formed by establishing the subordination of discourse to the control of normative authorities. The normative status of these authorities is achieved and made apparent through the description of their active role vis-à-vis service recipients. The normative authority “Social Affairs and Social Services Ministry” exists in order to “*treat/care for children and youths who are in distress*” (Reg. 8.9, p. 1). Actions under the Youth (Care and Supervision) Law of 1960 (Reg. 8.11) and even “*society in Israel*” (Reg. 8.6, p. 1) are described similarly. The normative authorities (“the ministry”, “the law”, “society”) are positioned as external to the “material body” of service recipients, and as having the privilege to make decisions about them and perform actions upon them. This solidifies their power to determine the actions to be performed by service providers, as agents mandated by normative authorities.

### Naming service recipients

Once the normative authorities have been positioned facing an object of reference, the form of service recipients themselves is established, first by giving them names and attaching meaning(s) thereto. Regulations name five groups of service recipients to which girls can belong to, each tied to specific conditions set forth in categories justifying the need for intervention on the part of normative authorities and their agents. Such categories explicitly reflect a connection between age and peril: **Child** (“*from birth to the age of 18… in distress*”; e.g., Reg. 8.9); **Minor** (who is “*under the age of 18*”; e.g., Reg. 8.11, p. 2), and to whom one of the following situations apply: “*there is no one responsible for him, the person responsible for him is unable to care for him, or neglects caring for or supervising him* […];” (Reg. 8.11, p. 2); **Teenagers and youths** (aged 14–25), who

*assemble in groups, were expelled from or dropped out of formal frameworks and loiter idly, and do not function or function with severe problems in adjustment, including asocial and criminal behavior. They mostly meet in the evening and nighttime, and belong to groups that evolve on their own in communities, against a background of non-functioning, feelings of deprivation, and rejection* (Reg. 3.22, p. 1-2);

**Teens harmed by addictions** (aged 12–24), whose “*routine functioning is damaged as a result of using drugs/alcohol/gambling, and who exhibit other and/or additional compulsive behaviors*” (Reg. 11.2, p. 2), and who “*uses […] and is characterized by physical and/or mental dependence*” (Reg. 11.2, p. 2); and **A girl or young woman** (aged 13–25), who is “*single […] whose behavior is characterized by self-destruction, and is deteriorating or in process of deterioration, and [who] experienced traumatic events, [either] mentally, emotionally, and socially*” (Reg. 17.1, p. 2). Delineating each group of service recipients to which the girl can belong implicitly involves, besides age and harmful situations, appearances of what each recipient, and the girl within her, is permitted or forbidden from being, if assistance is to be offered to her. Taking the definition of the “**girl or young women**” as an example, the reflection of what she is not allowed to be, is “married”. But, she is also “not allowed” to express self-protection, self-enhancement, or other positive behaviors, as the link between traumatic events and self-destruction must be fully observable for her to receive assistance.

### Distinguishing the various forms of service recipients within the service space

Once one “half” (normative authorities) of the described service space has gained the status of formative “truth”, and in order to coherently establish the service space, the “other half” (service recipients) correspondingly must be granted its own unique form of “truth” or existing form. This is done by applying various nuances to each group of service recipients to which the girl has to belong in order to receive assistance.

#### The emergent form of service recipient: Child

The terms repeated in regulations to describe children delineate the contours of a passive image. This service recipient is described as “*warded*”, and as “*subjected”* to neglect, violence, risk, and distress (Regs. 8.17, 8.34, 8.2, 8.5, and 8.9). Accordingly, the service space for children is consistently displayed as that in which the child “is placed”, “is supervised”, “is organized”, and “is treated”. Another explicit “truth” established regarding children stipulates that “*a child's growing up in his natural family ensures his proper development…”* (Reg. 8.17, p. 1). The use of the word “natural” implies that the image of “family” has a self-explanatory and inherent form, stemming from the most basic structures of humanity. Regarding development as “proper”, even without further explanation, implies that the “correct” way of developing can be measured and determined by normative authorities, and requires no elaboration, as it is granted the status of “truth” that precedes the discourse about children and families; as though “proper development” existed even before anyone defined it. Finally, the decisive premise that the result of growing up in a “*natural*” family is “proper development” is, once again, depicted as a “truth” that precedes discourse and is as such indisputable. If this is the case, what is to be “done” with children that do not grow up in their “natural” family? And what is there to learn about the family receiving services when development is not “proper”? Notably, the only form of children's families mentioned in regulations is a “natural family”. In accordance, services extended to children by normative authorities are all designed to imitate it: “*A foster family is the model closest to the natural family…*” (Reg. 8.2, p. 1); “*The center serves as a temporary substitute for the child's natural family*” (Reg. 8.2, p. 2).

#### The emergent form of service recipient: Minor

Beginning with the initial delimitation of minors' form based on their age, terminology used to describe minors who receive assistance from public social services is by way of negation. While children are at an age between birth and 18, a minor is “*whomever has not [yet] turned 18*” (Reg. 8.11, p. 13). Consistently throughout regulations, minors are defined by what is extraneous to them, leaving they themselves void of content. Moreover, their eligibility for assistance is determined through actions performed on them by others, e.g., their family abuses or neglects them. Regulations do not include any reference to forms of minors that reflect other possible facets of their being, besides their being described as “*needy*” (Reg. 8.11, p. 1) or “*a victim*” (Reg. 8.6, p. 1), thereby attaching essentialism to such traits among minors who receive services. As stated in regulations: “*The law specifies certain circumstances under which there is need for external intervention in order to protect the minor*” (Reg. 8.11, p. 1). Such circumstances become indisputable “truths” about minors' needs, that implicitly precede discourse about them, and are recognizable using absolute, unmistakable, fully generalizable conditions.

#### The emergent form of service recipient: Teenagers and youths

The borders of the forms of teenagers and youths receiving services, are established by using terms that illustrate their separation from other groups in society, a distinction that renders them inherently anomalous. Terms such as “*alienated*” and “*antisocial*” (Reg. 9.1, p. 1) position them at the margins of functional society. Facing them, and regarding the objectives of interventions with them, is the repeated use of the terms “*normative*” and “*education*” (Reg. 9.1, p. 2). These repeated terms perhaps indicate teenagers' and youths' ability to cease being “deviant” and reintegrate into society by exhibiting socially acceptable rules of behavior. Their “deviance” is often described using the term “*natural environment*” (e.g., Reg. 9.1), as opposed to “*problems*” and a “*phenomenon*” (e.g., Reg. 3.22). In other words, this form of service recipient removes herself from what is “natural” (thus existing with idiosyncratic consensual meaning, preceding the discourse that describes it) to others, and may be welcomed back into what is “normative” when exhibiting behaviors that enable “*reintegrating them into society*” (Reg. 3.22, p. 3).

The content of the designation “teenagers and youths” is described by the repeated use of active verbs, all depicting actions that are essentially negative: “*[engaging in] criminal behavior*”, “*loitering*”, “*[having] dropped out*” (Reg. 3.22). The repetition of various terms meaning “idleness” across regulations (e.g., Regs. 2.4, p. 60; 3.22, p. 1; 9.1, p. 1) stresses the fact that the image of these service recipients engages in actions considered useless. While the form of their image, unlike the form attached to “children” or “minors”, can contain their appearance as active figures, their activeness is delineated as categorically and absolutely useless and alienating, as a precondition for being considered eligible to receive services.

#### The emergent form of service recipient: Teens harmed by addictions

The outer contours of the form of the service recipient “teens harmed by addictions” is defined by describing various functions of its “material body”. Such functions, or actions, are depicted by both active verbs (e.g., “*using [drugs]*”) and passive language (e.g., “*to become addicted*”; Reg. 11.2). This phraseology reflects an internal contradiction in the form of these service recipients: On the one hand, they are victims of the problem, or the phenomenon, of addiction. On the other hand, they are implicitly blamed for taking the actions leading to their addiction. Accordingly, within the discourse space, as a condition for receiving services, the form of teens harmed by addictions who are eligible to enter the service space are expected to embody a paradox, and in this sense, the action engaged in by the body harms the same body that is also a passive victim of its own action. This duality enables the establishment of what is situated opposite this situation, i.e., what the normative authorities expect to achieve: “*gaining skills to cope [with the addiction]*”, and “*reintegrating into the normative trajectory of life*” (Reg. 11.2, p. 3).

#### The emergent form of service recipient: Girl or young woman

The borders of the form of the service recipient “girl or young woman” are delineated by the description of two spaces wherein her image is presented as plausibly located: one, indoors, and the other, outdoors. The division there between is marked by the repeated use of words such as “*circle*”, “*relationships*”, and “*home*” (e.g., Reg. 17.1). The outdoors, described in terms of impartially assessed realities, are established as “*crisis*”, “*danger*”, and “*risk*” (Reg. 17.1). The indoor space is described as emotional and experiential, by using words such as “*emotions*”, “*stress*”, “*support*”, and “*belonging*” (Reg. 17.1). Accordingly, the external, perceived as an objective evaluation of girls“ and young women's states, obtains the status of “truth”, while the internal is inter-subjective, “soft”, and deriving from individual experiences. In the external space, the form of this service recipient is imagined to be passive and vulnerable in all areas of her life: “*a girl and young woman who was or is a victim of sexual abuse, a victim of violence in and outside the family*” (Reg. 17.1, p. 2). Her vulnerability is essential and overwhelming, and she is, throughout most regulations “*needy*” of “*protection*”, “*support*”, and “*empowerment*”. Others know the truth about her, make decisions for her, and treat her.

However, regarding the inner space, while her image obtains the form of an active agent, her actions place her at continuous risk: “*a girl or young woman […] whose behavior is characterized by self-destruction*” (Reg. 17.1, p. 2). According to this formula, her passiveness, as interpreted by others, holds the evident key to her protection, while her actions, which derive from her own decisions and interpretations, threaten her.

The “truth” about girls and young women eligible for assistance is established as follows: “*Among teenaged girls and young women there is a phenomenon wherein some have difficulties fulfilling the roles that are acceptable and typical for their age, as a result of their exposure to hardships in the family and in society*” (Reg. 17.1, p. 1). This solidifies expectations of girls (and young women) in Israel as preceding the discourse about them. In this “reality”, the source of the “difficulty in fulfilling roles” is an absence of ability, or deprivation: “*These girls and young women are deprived emotionally and functionally, and often lack the capacity to forge stable bonds with their close environment*” (Reg. 17.1, p. 1). Here, what is “lacking” implies the existence of a “whole”, and the “absence” stands opposite an unwritten “presence” that obtains a self-evident status of natural “fact”. Who, then, is the girl who is eligible to become a service recipient? She is whomever her hypothetical normative counterpart, is not.

### Pinning the figure of the service recipient down to its preassigned form

Thus far, we have seen how the discourse surrounding “girls in distress” in regulations includes the subjugation of groups of service recipients to which she may belong, to the normative authorities controlling the discourse surrounding the service space. The next stage then becomes the scientific classification of working with girls as an unquestioned discipline. Two elements of this process have already been displayed: By presenting assumptions about service recipients and their lives as “natural” truths that precede the discourse dictated by normative authorities; and by repeating them again and again, enabling discourse to take on the appearance of previous meaning, connected to common knowledge. These two maneuvers delimit groups of service recipients and designate coherent forms for identifying them according to a repeated internal logic. This fulfills Foucault's (5, 6) principle of the author: There is now a speaker of the discourse, with the legitimacy, knowledge, and power to shape services, to dictate the actions of their providers/agents, and to mark the acceptable borders of their recipients' forms.

For scientific discipline to be fully realized and services to be made available, these elements of the discourse must be tied *via* discourse to its subjects' individual particular identities.

For the service recipient “**Child**”, this is done by repeating the phrase “*the child and the family*” in regulations (e.g., Reg. 8.9, p. 5), i.e., the child and her family are constantly presented as a single unit. When the family cannot be, or is not, what is acknowledged in the discourse as acceptable, the service fulfills its part of the symbiosis with the child, and the discourse shaping the service space is tied to the child's own particular identity. The service recipient “**Minor**”, is defined, as aforementioned, through what it is not, automatically leaving a void to be filled by the dominant discourse, that is tied to minors' particular identity as it marks all that lies beyond them. Also, regulations regarding minors establish the constantly crucial involvement of the scientific discipline and its agents in the life of the minor receiving services, while pointing to her as being in a perpetual state of acute crisis (“*the social worker must be housebound, or carry a mobile communications device outside, ready to respond immediately to any call regarding a minor in need*”; Reg. 8.27, p. 2).

“**Teenagers and youths**” are tied to their own particular identities within the discourse established by normative authorities through the continuous use of the term “framework” (e.g., “*educational frameworks*”, “*formal frameworks*”; Reg. 3.22). These frameworks can perhaps be analogized to a picture frame or a window frame, i.e., a mold that protects the edges of something, defines its borders, and is mostly inseparable from its familiar image. Accordingly, the frameworks in which services are provided are part and parcel of the discourse, and become what holds the form together. Another way this is done is by describing teenagers and youths as a social group with its own unique lifestyle that is idiosyncratic to it, in phraseology that is almost anthropological or zoological: “*Follow the times and area in which this population dwells, learn its ways of recreation and behavior…*” (Reg. 2.4, p. 60); “*It is the role [of the youth social worker] to go out to the population's natural habitat, in its own hours and time*” (Reg. 2.4, p. 60). The form of the image “**teens harmed by addictions**” is tied to the internal logic of splitting it, by external discourse, into the aforementioned paradox, i.e., between the girl that is both to blame and a victim of her own addiction, “savable” only by way of external intervention, without which she is presumably unable to create a separation between herself and “*the phenomenon*” (Reg. 11.2, p. 1), and is doomed to be trapped therein forever.

Finally, the form of the image “**girl and young woman”** is solidified and tied to her particular identity through repeated reference to the theme of “cycles of distress”. For example: “*Removing the girl or the young woman from the cycles of sexual abuse and violence*” (Reg. 17.1, p. 3); “*Removing […] from the circles of […] distress..*.” (Reg. 17.4, p. 2), and “*The responses are based on the unique needs […] for removing the girls from the circles of violence*” (Reg. 17.1, p. 4). This decision depicts the risks posed to the girl as necessarily endless, correctible only through her removal by external authorities. As aforementioned, this state of affairs is created by the behavior of the girl herself: “*a girl or young woman that was/is a victim […] and [who] employs one or more of the following behaviors…*” (Reg. 17.1, p. 2). The word “*and*” implies that to obtain assistance, the girl must fulfill not only the condition of victimization, but also exhibit self-harm. This framing restricts girls who were abused and need assistance to a victimhood < > guilt cycle, again disruptable only by external intervention. Interestingly, this intervention entails not only her removal from one circle/cycle, but also her placement in alternative circles/cycles, preselected for the girl by normative authorities: “*[treatment in the transitional home is aimed at] managing proper relationships in all the circles to which the girl belongs*” (Reg. 17.2, p. 2). In this sense, the decisive change that the girl receiving services can hope for, the only future foreseen for her within the particular identity that she must adopt as a condition for receiving services, is moving from circles in which she is the object of abuse, to circles wherein she is the object of protection.

### The “birth” of the coherent image of the “girl in distress” and the performative preconditions for her receiving assistance

Now that the distinct, coherent forms of each group of service recipients to which the girl may belong to has been determined, it is possible to examine the qualities of the image of the “girl in distress” that is present in all of them, and that is the ultimate object of public social services provided to girls. Binding together splinters of the girl's form scattered across regulations, common threads that run through regulations reveal themselves, as they point to the performative acts in which she is expected to engage and perpetuate in order for her to be professionally comprehensible and receive assistance.

The first thread underscores her distinction from boys receiving services. In regulations wherein girls and boys are addressed separately, the girl is typically “*treated*” and “*removed from*” (e.g., Reg. 17.4), while the boy (or even the seldom-used “boy/girl”) “*signs*”, and “*takes*” (e.g., Reg. 11.2). These differing associations with activity vs. passivity are far more than semantic. They delimit the “girl in distress” in ways that restrict her activity, portraying her as the proverbial weak, unintelligent “damsel in distress”. In order to be accepted as a recipient of services by normative authorities, she must perform accordingly. At the same time, unlike descriptions of services provided only to boys, some services for girls are designed to instill “*skills that will enable her to maintain a relationship with the opposite sex […]*” (Reg. 17.1, p. 2–3). Here, the gender of the girl is equated with exclusive responsibility for relationships with boys and their sexually-particular “material bodies”, possibly implying guilt when such relationships turn against her. They also establish a binary gender conceptualization wherein the “natural” is predetermined as exclusive attraction to the “opposite sex”.

The second has to do with the repeated restriction of the “girl in distress” to domestic spaces of existence. Assistance to service recipients who may be “girls in distress” are denoted using words closely associated with this space, for example: “*a welcoming home*”, “*a transitional residence*”, or a shelter, described as “*a private home in a residential neighborhood*” (e.g., Reg. 17.3).

Conclusively, from the establishment of normative authorities, through the denoting of the various forms of service recipients within the service space, followed by the tying of these forms to recipients' particular identities, and ending with determining performative acts expected of “girls in distress” in order for them to receive public social services, what at first appears to be a simple description of girls granted assistance, can be viewed as none other than an object created by discourse, with or without taking its/her actual individual identities, voice, or circumstances into account. This object, constantly placed on the dichotomous independence/guilt < > dependence/victimhood track, is eventually “redeemed” only when (re)embedded in the performative acts expected of its “normative' counterpart: “*building a proper relationship and positive communication with members of her family; strengthening her ability to develop normative social bonds in accordance with her age […] encouraging her integration into normative formal and social frameworks*” (Reg. 17.1, p. 3).

## Discussion

The results of our interpretative analysis shed light on a systematic process of constructing the image of the “girl in distress” in regulations guiding public social services offered to her. They reveal how the language and terminology used in regulations can be viewed as a tool molding her form as weak, vulnerable, irrational, and perpetually troubled, yet “guilty” of actions that render her eligible for assistance. As is common in interpretative works, some of the analysis and the conceptualization of its results are intertwined in the presentation of the findings. We seek, however, to focus the discussion on three issues consistently appearing in regulations, the discourse reflected therein, and the insights that can be drawn about this discourse and its applications in terms of epistemic in/justice; and to address some of their possible meanings and implications.

The first issue is the repeated representation, description, and reference to “girls in distress” as passive, voiceless, individuals. The image of the “girl in distress” appears in regulations *ex nihilo*, as a formative silhouette structured only of conditions that she must fulfill in order for her distress to become apparent to others. If she fails to perform in compliance therewith, she will be rejected by normative authorities as not in “real” need of assistance, just as was plausibly done to her in other cultural or social spaces, following her failure to behave as is expected of girls her age. The “girl in distress's” distress, then, is not enough for her to turn visible: She must maintain the image determined for her, or else she will be left to fend for herself. Any experiences, individual identities, knowledge by experience or narratives that stray from this image and the contours determined for its form, are implicitly structured as irrelevant to the process of assisting or treating her. This, suggestibly constitutes a radical appearance of epistemic injustice, less discussed in mainstream literature. While, as noted earlier in this article, a common conceptualization of epistemic injustices groups them into either testimonial or hermeneutical injustices (13), the processes uncovered in the present study seem to echo what can be regarded as “existential epistemic injustice”. This sort of epistemic injustice does not involve the willingness neither the capacity of listeners to accept the knowledge and experiences put forward by oppressed populations as legitimate and trustworthy (as would require the correction of testimonial injustice); nor does it pertain to societal or organizational skills to comprehend certain groups' social experiences due to “prejudicial flaws in shared resources for social interpretation” [s the correction of hermeneutical injustices would demand; (13), p. 148]. Rather, existential epistemic injustice is the state of negating not only the credibility of the speaker or the unique character of their narrative, but nullifying the very virtue of their existence as speakers. Existential epistemic injustice, thus addresses processes and mechanisms often hidden deep in the nuances of discourse, subjugation and inscription, which create a clear distinction between forms of speakers who are more or less victimized by epistemic hierarchies, and those rendered essentially invisible in the struggle for epistemic privilege and recognition. The image of the “girl in distress” that emerges from our analysis has her concrete, individual, experiential existence placed under question. If she does not express her distress in ways stipulated as preconditions for receiving assistance or treatment, she remains external to the arena where discourse is played out. Her voice does not reach the stage of being discriminated against; it is substituted with silence. This trajectory of epistemic violence (29) makes identifying testimonial or hermeneutical epistemic injustice all the more difficult. The distress that is not identifiable by normative authorities is situated beyond the structure designated to treat it. We propose that when this occurs, the girl's solitude is analogous with the empty space that her human image leaves behind in Israeli society, as she may continue to retreat into areas of hardship outside of most people's fields of vision.

In Butler's critique of Foucault, she wrote: “Although Foucault writes that the body is not stable and cannot serve as a common identity among individuals cross-culturally or transhistorically, he nevertheless points to the constancy of cultural inscription…” [(30), p. 604]. In this vein, according to her, while Foucault acknowledges that morality, sex, and other elements considered “natural” are in fact the result of cultural inscriptions on the body, he accepts the “material body” itself as a neutral platform that exists prior to the discourse and prior to cultural inscription. Butler proposed that even the coherence of the body is not independent of inscriptions. It follows, then, that the binary division, apparent in regulations, between actions associated with (and thus acceptable for) girls and those expected of boys, constitute a powerful inscription mechanism. The active body of girls receiving services is coherent only when performing the actions considered reasonable for it. If the girl is to be comprehensible to normative authorities, she must maintain the coherent image that authorities delimit for her.

Foucault (8) cited ways in which power is involved in the categorization of individual characteristics as “deviant”. The “deviant” must thus be classified as such, and inspection thereof is justified as key to the maintenance of the social order. In the regulations that we analyzed, “girls in distress” are presented inseparably from the deviance attached to them. This deviance, both when described as needy passivity and as self-destructive activity, has the same end result: The girl must relinquish control to normative authorities over deciding what is best for her.

The second issue apparent throughout regulations has to do with the confinement of the girl to the physical or figurative domestic space. While the literature and direct experiences of woman show that for many girls, the meaning attached to the home portrays it as unsafe, sometimes even more threatening than other environments (31), the discourse found in regulations treats it differently: Solutions to the problems of “girl in distress” are widely described in terms referring to the home, or even designed as homes. In this sense, “home” becomes an essential element of the girl, outside of which her presence is not even imagined. Accordingly, using the process described by Foucault (7), the power of normative authorities operates on the individual that is the girl by intervening in her life, studying her, and encouraging her to become an “oppressable” individual by fostering her “particularity” and tying her thereto: The girl belongs in the home, and anything beyond it is outside of her reach.

Finally, throughout the regulations, the image and form delimited for the “girl in distress” depicts her as void of any national, cultural, or ethnic characteristics. Gender, age, and risk thus become the only “real” traits relevant to providing effective assistance to her. This could, in a way, be seen as a generator of equity, as all girls addressed in regulations are treated the same, regardless of their backgrounds. However, at the same time, the discourse reflected in regulations is blind to some of the most central identity, personal, and familial aspects of girls' lives. This stands out especially in the diverse, fraught, and conflictual context of Israeli society, so deeply entrenched in institutionalized gaps (32). Arguably, this exemplifies what can be referred to as oppression-by-dismissal or obscuring oppression (33), veiling structural factors frequently contributing to the discrimination and vulnerability that so many girls in Israel face (34). This issue is pivotal and deserves dedicated, in-depth discussion and interpretation, currently already taking place.

Some of the limitations of the present study should be taken into account when reviewing its findings. Firstly, as in all deductive analyses, our insights reflect the results of our own interpretations and readings into the theoretical principles applied. While this is organic to the paradigmatic approach to the present research, it is worthy of consideration. Secondly, while we believe that the understandings provided in this article can be applicable to a wide range of situations, it is left up to the reader to determine if and how they translate into her/his own specific context.

As a closing remark, it is important to note that although the results of our analysis and the interpretation thereof are presented as (and are in actuality) critical toward policymakers and the agents of their authority, they at the same time offer room for optimism. While policy must—though debatably—somehow mark who is and who is not eligible for services, this also gives the powers that design it the option of using discourse to rectify social injustices. Our suggestions also do not disregard broad facets of the milieu in which services to girls are provided. Providers of assistance and treatment to girls are, in a way, in some cases subjected to the very same discourse exposed in the present study. Categorically grouping them together with the elites dominating discourses flattens potential discussions on methods and allies in the movement toward shattering the shackles of oppression.

## Data availability statement

Publicly available datasets were analyzed in this study. This data can be found here: https://www.gov.il/he/departments/policies/molsa-social-regulations.

## Author contributions

LL and MC contributed to conception and design of the study, lead the analysis of the data, and wrote the first draft of the manuscript. RA, SP, and HK-E contributed to later drafts and advanced conceptualizations. The manuscript's submitted version was read and approved by all authors.

## Funding

This research was supported by a grant from the Ministry of Science and Technology.

## Conflict of interest

The authors declare that the research was conducted in the absence of any commercial or financial relationships that could be construed as a potential conflict of interest.

## Publisher's note

All claims expressed in this article are solely those of the authors and do not necessarily represent those of their affiliated organizations, or those of the publisher, the editors and the reviewers. Any product that may be evaluated in this article, or claim that may be made by its manufacturer, is not guaranteed or endorsed by the publisher.
